# Autochthonous Chikungunya Transmission and Extreme Climate Events in Southern France

**DOI:** 10.1371/journal.pntd.0003854

**Published:** 2015-06-16

**Authors:** David Roiz, Philippe Boussès, Frédéric Simard, Christophe Paupy, Didier Fontenille

**Affiliations:** 1 MIVEGEC (Infectious Diseases and Vectors: Ecology, Genetics, Evolution and Control), UMR IRD224-CNRS5290-UM, Institut de Recherche pour le Développement (IRD), BP 64501, Montpellier, France; 2 Institut Pasteur du Cambodge, Phnom Penh, Cambodia; Oswaldo Cruz Foundation, BRAZIL

## Abstract

**Background:**

Extreme precipitation events are increasing as a result of ongoing global warming, but controversy surrounds the relationship between flooding and mosquito-borne diseases. A common view among the scientific community and public health officers is that heavy rainfalls have a flushing effect on breeding sites, which negatively affects vector populations, thereby diminishing disease transmission. During 2014 in Montpellier, France, there were at least 11 autochthonous cases of chikungunya caused by the invasive tiger mosquito *Aedes albopictus* in the vicinity of an imported case. We show that an extreme rainfall event increased and extended the abundance of the disease vector *Ae. albopictus*, hence the period of autochthonous transmission of chikungunya.

**Methodology/Principal Findings:**

We report results from close monitoring of the adult and egg population of the chikungunya vector *Ae. albopictus* through weekly sampling over the entire mosquito breeding season, which revealed an unexpected pattern. Statistical analysis of the seasonal dynamics of female abundance in relation to climatic factors showed that these relationships changed after the heavy rainfall event. Before the inundations, accumulated temperatures are the most important variable predicting *Ae. albopictus* seasonal dynamics. However, after the inundations, accumulated rainfall over the 4 weeks prior to capture predicts the seasonal dynamics of this species and extension of the transmission period.

**Conclusions/Significance:**

Our empirical data suggests that heavy rainfall events did increase the risk of arbovirus transmission in Southern France in 2014 by favouring a rapid rise in abundance of vector mosquitoes. Further studies should now confirm these results in different ecological contexts, so that the impact of global change and extreme climatic events on mosquito population dynamics and the risk of disease transmission can be adequately understood.

## Author Summary

During last years, we have seen an astonishing expansion of Chikungunya virus and an increase in dengue cases worldwide, together with the worldwide expansion of the Asian tiger mosquito *Aedes albopictus*. In addition, extreme rainfall events are envisaged to become increasingly likely as a result of ongoing climate change, but controversy surrounds the relationship between extreme rainfall events and mosquito-borne diseases. The common view in most works on climate and mosquito-borne diseases is that heavy rainfalls produce a flushing effect of immature mosquitoes in breeding containers, diminishing the mosquito abundance and in turn diminishing disease transmission. We analysed the relationships between the autochthonous chikungunya transmission in Montpellier (Southern France) in 2014, an extreme rainfall event that flooded the city, and a close monitoring of the vector *Ae*. *albopictus*, revealing an unexpected pattern. This extreme rainfall event did not, in fact, decrease but instead had increased the global risk of chikungunya transmission by sustaining high abundance of the disease vector *Ae*. *albopictus*, hence extending the transmission period. We propose that an effort on source reduction campaigns must be implemented after heavy rainfall events. These results are relevant to those involved in the surveillance and control of chikungunya and dengue transmission in temperate as well as tropical areas.

## Introduction

Extreme precipitation events are envisaged to increase as a result of ongoing global warming [1]. Heavy rains have consequences for infectious diseases, with, in particular, some vector-borne disease outbreaks being associated with flooding [2,3]. However, the relationships between flooding and vector-borne diseases are mired in controversy and need to be clarified [4].

A common belief among the scientific community and public health officers is that “*heavy rainfalls produce a flushing effect of immature mosquitoes (larvae and pupae) in breeding containers*, *diminishing the mosquito abundance*” [3,5], in turn diminishing disease transmission. The common view in most works on climate and mosquito-borne diseases is that “*Intense rainfall may wash out breeding sites and thus have a negative effect on vector populations*” [6].

During September-November 2014, French health authorities reported a cluster of 11 autochthonous cases of chikungunya in the city of Montpellier in the vicinity of a recently imported case [7, 8]. This was the first report of locally transmitted chikungunya in France since 2010, adding to the 4 cases of dengue reported earlier that year in the neighbouring PACA region, and dengue cases in 2010 and 2013 [7–9]. Abundances of the tiger mosquito *Aedes albopictus*, the competent disease vector, in an increasing number of places and the large number of imported cases of chikungunya (443 cases), dengue (163 cases) and co-infections (6 cases) in France [7], did indeed concur to increase the risk of autochthonous transmission in Southern France.

The objective of this study was to analyse the influence of climatic variables, including an extreme rainfall event, on *Ae*. *albopictus* abundance in Montpellier in 2014, and its impact on the risk of autochthonous chikungunya transmission.

## Methods

Mosquitoes were sampled in the municipalities of Montpellier and Castelnau-le-Lez in the Province of Hérault, region Languedoc-Roussillon, southern France, with a Mediterranean climate and a resident human population of 400.470 inhabitants spread over 14.62 Km^2^.

Adult mosquitoes were collected using 24 BG-Sentinel traps (BioGents, Regensburg, Germany), with the attractant BG lure, a synthetic lure developed to mimic human odors, consisting of lactic acid, ammonia, and caproic acid on a long-lasting lure. Traps were located in 8 sampling sites separated by a mean geographic distance of 2.2 Km. Each sampling site consisted of a private house with garden, and three trap replicates set 5–10 m apart were operated in each site. Each trap was connected to a battery (12 V), and the captures were conducted for 24 H, once a week, from the 21th week (2nd week of May) to the 50th week (2nd week of December) of 2014. Sampled mosquitoes were transported live to the laboratory in an insulated thermal bag filled with ice and frozen at -20°C. The species and sex of each individual were identified in the laboratory with a stereomicroscope using the appropriate taxonomic keys [10]. Although several species were captured, including *Ae*. *albopictus*, *Culex pipiens*, *Culiseta longiareolata*, *Aedes caspius* and *Anopheles maculipennis s*.*l*., only *Ae*. *albopictus* females were included in the analysis. The mean weekly abundance of *Ae*. *albopictus* females captured in the 24 traps was used as the dependent variable.

In parallell, eggs of *Ae*. *albopictus* were collected using oviposition traps (ovitraps) placed in each of the 24 locations where the adult population was monitored. *Bacilus thuringiensis var*. *israeliensis* granules (VectoBac, ValentBioSciences) were added to the water to prevent larval development and traps were checked once a week. Eggs were counted under a stereomicroscope and averaged across traps and over collection sites.

Daily mean, minimum and maximum air temperatures, daily total precipitation and insolation time for the study period were obtained from the Montpellier Fréjorgues meteorological station (http://www.meteociel.fr/climatologie/villes.php?code=7643&mois=6&annee=2014), located 6 Km from the city centre. For the purposes of our study, these data were pooled by week. Then, for temperature, weekly means and weekly range were computed, and for rainfall, total weekly precipitation was calculated. Environmental variables may have a strong influence on mosquito populations not only at the time of adult capture but also, and mainly, at egg-laying and larval development which occur 1–4 weeks earlier. Therefore, accumulated temperature and rainfall were calculated over the 4 weeks preceding the week of sampling. Growing Degree Days (GDD) were calculated with a baseline temperature of 11°C to compute weekly accumulated Growing Degree Days (GDD). Based on our previous work, we also assessed a ‘bounded’ estimate of the accumulated GDD (Bounded accumulated GDD), with a maximum threshold of 1350 accumulated GDD above which any further increase in GDD is considered detrimental and counted negatively [11, 12].

We developed a Generalized Linear Model with negative binomial distribution (as the data were over-dispersed) to investigate whether *Ae*. *albopictus* adult female abundance was influenced by temperature and rainfall before and after the extreme rainfall event. The response variable was the total weekly *Ae*. *albopictus* adult female abundance, and the selected explanatory variables were the average weekly temperatures (minimum, mean and maximum), the total weekly precipitation, all the accumulated temperature and rainfall variables (see Climatic data section for details), Weekly Growing Degree Days, Accumulated Growing Degree Days and Bounded Accumulated Growing Degree Days. Due to the highly expected co-linearity between the explanatory variables, different univariate models were built and the best model was selected on the basis of AIC (Akaike Information Criterion), ΔAIC and Akaike weights. We calculated the explained deviance as: (Null deviance-Residual deviance)/ Null deviance. Statistical analysis was performed in R version 2.14.2 [13] with the packages MASS [14] and MuMin[15].

## Results

**Fig 1:**
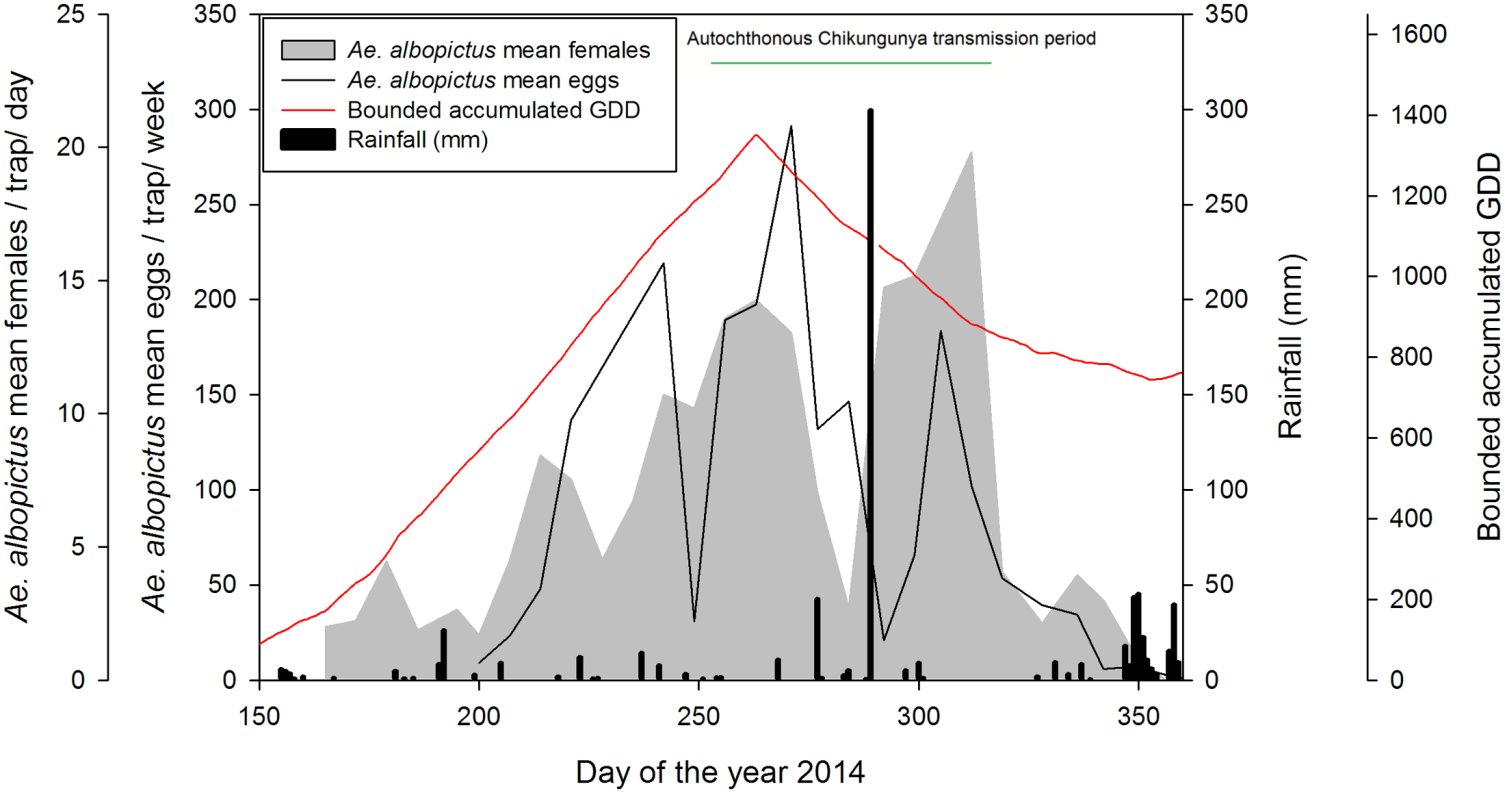
Mean number of *Ae*. *albopictus* eggs per trap per week (black line), *Ae*. *albopictus* females per trap per day (grey area) in 24 BGs and 24 ovitraps positioned in 8 locations in Montpellier in 2014. Autochthonous chikungunya transmission period (green line); weekly bounded accumulated Growing Degree Days (red line); weekly rainfall (black bars).

**Fig 2:**
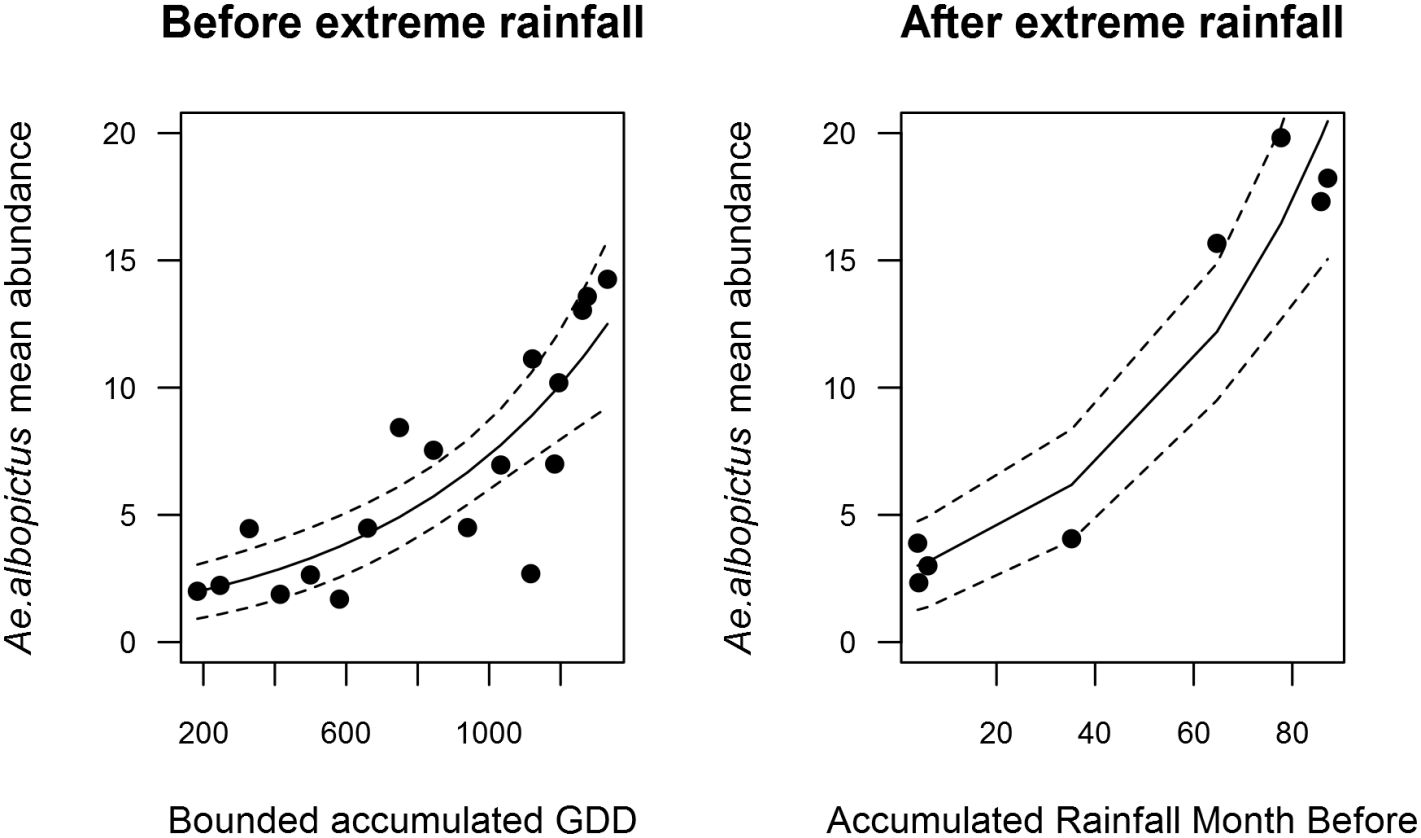
Results from the Generalized Linear Model with negative binomial distribution showing the impact of climatic variables on *Ae*. *albopictus* abundances before (left panel) and after (right panel) the extreme precipitation event in 2014. Before the inundations, weekly bounded accumulated Growing Degree Days were related to vector abundance. After the inundations, accumulated rainfall is more important than temperature (see also Table 1).

We developed a close monitoring of the adult population of the chikungunya vector *Ae*. *albopictus* in Montpellier through weekly sampling over the entire mosquito breeding season (*i*.*e*., May to November 2014). Although mosquito densities steadily declined after peaking in late August, extreme rainfall events flooding the area at the end of September and beginning of October (Week 39), with up to 252 mm of rain falling in just 3 hours (recorded on 29th September in Montpellier), resulted in an explosive mosquito population growth extending into October and surpassing the abundance peak recorded earlier in August (Fig 1). Statistical analysis (Table 1; Fig 2) revealed that, before the inundations, temperature (Accumulated Growing Degree Days) was the most important variable predicting the seasonal dynamics of *Ae*. *albopictus*, with 69.3% of the variance explained. However, after the inundations, accumulated rainfall over the 4 weeks before capture predicted *Ae*. *albopictus* seasonal dynamics, explaining 92.3% of the variance (Table 2; Fig 2). The seasonal pattern of eggs abundance in the ovitraps closely matched that of female abundance, with a lag of several days (Fig 1).

**Table 1:** Results of the model selection of the Generalized Linear Model with negative binomial distribution showing the effect of climatic variables on *Ae*. *albopictus* abundances before the inundation in 2014.

Variable	AICc	delta	weight
Bounded accumulated GDD	86.20	0.00	0.99
Accumulated GDD	95.37	9.17	0.01
Accumulated temperatures one month before	99.12	12.92	0.00
Maximum weekly temperatures	103.41	17.21	0.00
Accumulated temperatures two weeks before	103.66	17.46	0.00
Accumulated rainfall two weeks before	104.30	18.10	0.00
Growing degree days (GDD)	105.04	18.83	0.00
Mean weekly temperature	105.04	18.83	0.00
Weekly rainfall	105.65	19.45	0.00
Weekly insolation time	105.95	19.74	0.00
Accumulated rainfall one week before	106.12	19.92	0.00
Accumulated temperature one week before	106.33	20.13	0.00
Weekly minimal temperatures	106.49	20.28	0.00
Accumulated rainfall four weeks before	107.07	20.86	0.00

The best model explains 69.3% of the variance.

**Table 2:** Results of the model selection of the Generalized Linear Model with negative binomial distribution showing the effect of climatic variables on *Ae*. *albopictus* abundances after the inundation in 2014.

Variable	AICc	delta	weight
Accumulated rainfall four weeks before	46.54	0.00	0.60
Maximum weekly temperatures	48.35	1.81	0.24
Accumulated temperatures two weeks before	50.11	3.57	0.10
Accumulated temperature one week before	53.34	6.81	0.02
Mean weekly temperature	54.01	7.47	0.01
Growing degree days (GDD)	55.08	8.54	0.01
Accumulated GDD	55.67	9.14	0.01
Accumulated temperatures one month before	56.03	9.50	0.01
Weekly minimal temperatures	58.19	11.66	0.00
Bounded accumulated GDD	58.68	12.15	0.00
Weekly insolation time	61.78	15.25	0.00
Accumulated rainfall two weeks before	62.45	15.92	0.00
Accumulated rainfall one week before	64.40	17.86	0.00
Weekly rainfall	64.88	18.34	0.00

The best model explains 92.3% of the variance.

## Discussion

We have shown that the extreme precipitation event clearly contributed to increasing and extending the abundance of the disease vector *Ae*. *albopictus*, and hence to extending the period of autochthonous transmission of chikungunya in Montpellier in 2014. Female density increased rapidly after the extreme event, soon followed by a rise in the number of eggs collected in ovitraps. Therefore, and contrary to common belief [3,4,5,6], our empirical data suggest that heavy rainfall events do not, in fact, decrease but instead may increase the global risk of chikungunya (and other arboviruses) transmission, by extending the transmission season. This is the first evidence to support the relationship between heavy rainfall and chikungunya transmission. Our observations before and after the extreme precipitation event, suggest that heavy rains after a dry period with low precipitations have filled all the peridomestic containers where desiccated eggs of *Ae*. *albopictus* were to be found, and that it was this situation which gave rise to the increase in mosquito numbers several weeks later. Indeed, the majority of breeding sites colonized by *Ae*. *albopictus* larvae in Mediterranean Europe are small peridomestic containers of less than 10 L. such as scuppers, flowerpot saucers, drums, buckets, solid waste and others, whereas the productivity of catch basins is generally much lower [16, 17, 18]. In Southern France, more than 80% of breeding sites are situated in the private domain and only 12% of the immature stages were detected in catch basins [16]. The outcome of such extreme climatic events might therefore be different in different ecological contexts, depending on the larval ecology of the species. For example, West Nile virus (WNV) outbreaks have been associated with flooding, in Romania, the Czech Republic and Russia [19, 20, 21]. However, these outbreaks were related to flooded building basements, resulting in an increase in populations of the WNV vector *Culex pipiens*, which thrives in large water collections that are not suitable for *Ae*. *albopictus* development. Further studies are needed to explore the general relevance of our findings and their implications for diseases transmission by *Aedes albopictus* in Europe, as well as elsewhere where the species has established.

Because our data showed that heavy rains in Mediterranean cities impacted on *Ae*. *albopictus* abundances, we propose that an effort on source reduction campaigns must be implemented after such heavy rainfall event. In fact, these high-volume rainfall events, referred to as *Cévenol episodes*, are relatively frequent in the south of France.

We further encourage tropical countries that are endemic for Chikungunya and Dengue to develop similar studies in order to explore the relevance of enforcing source reduction approaches following extreme climatic events and we believe that our results should be brought to the attention of those involved in the surveillance and control of vectors and vector-borne diseases in the context of global change in temperate as well as tropical areas of the world.
